# Полиморфизм гена маннозосвязывающего лектина у коренных популяций территорий Арктической зоны Российской Федерации

**DOI:** 10.18699/VJ20.685

**Published:** 2020-12

**Authors:** S.Yu. Tereshchenko, M.V. Smolnikova

**Affiliations:** Scientific Research Institute of Medical Problems of the North, Federal Research Center ”Krasnoyarsk Science Center“ of the Siberian Branch of the Russian Academy of Sciences, Krasnoyarsk, Russia; Scientific Research Institute of Medical Problems of the North, Federal Research Center ”Krasnoyarsk Science Center“ of the Siberian Branch of the Russian Academy of Sciences, Krasnoyarsk, Russia

**Keywords:** MBL2, gene polymorphism, newborns, Russia, Arctic populations, MBL2, полиморфизм генов, новорожденные, Россия, арктические популяции

## Abstract

Маннозосвязывающий лектин (mannose-binding lectin, MBL) – паттерн-распознающий острофазовый белок, относящийся к системе врожденного иммунитета и активно участвующий в элиминации широкого круга патогенных микроорганизмов посредством активации лектинового пути системы комплемента. Значительная часть человеческой популяции имеет врожденно низкий уровень продукции и/или низкую функциональную активность MBL вследствие носительства различных вариантов гена MBL2, что может модифицировать течение самых разнообразных инфекционных заболеваний. Частота генотипов и гаплотипов полиморфизмов в гене MBL2 имеет значительные популяционные различия. К настоящему времени данные относительно распределения генотипов гена MBL2 в коренных популяциях территорий Арктической зоны Российской Федерации отсутствуют. Цель исследования – изучение частоты и этнической специфики распределения аллельных вариантов полиморфизмов гена MBL2 rs11003125, rs7096206, rs7095891, rs5030737, rs1800450 и rs1800451 и их гаплотипов в популяциях Таймырского Долгано-Ненецкого района Красноярского края (ненцы, долганы-нганасаны, русские). В настоящем исследовании нами впервые получены данные о частотах генотипов и гаплотипов гена MBL2 у коренных народностей, проживающих на территориях Арктической зоны Российской Федерации. Частота встречаемости гаплотипа HYPA, ассоциированного с высокой концентрацией MBL, составила 35.4 % для русских новорожденных Восточной Сибири, что соответствует частотам европейских популяций (27–33 %). У новорожденных арктических популяций частота гаплотипа HYPA была статистически значимо выше, чем у русских, и составила 64 % для ненцев и 56 % для долган-нганасан, что приближается к значениям частот, выявленных для эскимосов и североамериканских индейцев (64–81 %). Популяции ненцев и долган-нганасан демонстрируют существенно более низкие частоты MBL-дефицитных гаплотипов в сравнении с европеоидами Восточной Сибири (3.9, 6.4 и 21.3 % соответственно). Мы предполагаем, что изолированные арктические популяции исторически позже столкнулись с некоторыми внутриклеточными инфекциями (туберкулезом, лепрой) и, в отличие от европеоидных популяций, сохранили сформированную на ранних этапах эволюции человека высокую активность лектинового пути активации комплемента.

## Abstract (en)

Mannose-binding lectin (MBL) is a pattern recognizing acute-phase protein of the innate immunity system actively involved in the elimination of a wide range of pathogenic microorganisms by activating the lectin pathway of the complement system. A significant part of the human population has a congenitally low production level and/or low MBL activity due to the carriage of various MBL2 variants, which can modify the course of a wide range of infectious diseases. The genotype and haplotype frequencies of the MBL2 polymorphisms have significant population differences. So far, data on the prevalence of the MBL2 genotypes in indigenous populations of the Russian Arctic regions have not been available. The aim of the study was to analyze the frequency and ethnic specificity of the distribution of allelic variants of the MBL2 polymorphisms rs11003125, rs7096206, rs7095891, rs5030737, rs1800450 and rs1800451 and their haplotypes in the populations of the Taimyr Dolgans-Nenets region of the Krasnoyarsk territory (Nenets, Dolgans-Nganasans, Russians). Data on the genotype and haplotype frequencies of the MBL2 gene among indigenous peoples of the Russian Arctic territories was first obtained in the study. The HYPA haplotype prevalence associated with a high concentration of MBL amounted to 35.4 % for Russian newborns in Eastern Siberia, corresponding to the one for European populations (27–33 %). In newborns of the Arctic populations, the prevalence of HYPA haplotype was significantly higher than in Russians and amounted to 64 % for Nenets and 56 % for the DolgansNganasans, which is close to the one detected for the Eskimos and North American Indians (64–81 %). Populations of Nenets and Dolgans-Nganasans demonstrated a significantly lower prevalence of MBL-deficient haplotypes compared with Caucasians of Eastern Siberia (3.9, 6.4 and 21.3 % respectively). Isolated Arctic populations were suggested to experience some intracellular infections (tuberculosis, leprosy) historically later and, unlike Caucasoid populations, to retain the high activity of the lectin complement activation pathway formed in the early stages of human evolution.

## Введение

Система комплемента – древнейший компонент врожденного иммунитета, основной функцией которого является преимущественно интраваскулярная элиминация бактериальных агентов. Кроме того, протеины комплемента играют роль своеобразного моста между системами врожденного и адаптивного иммунитета, обеспечивая адекватные условия для созревания и дифференциации Ви Т-лимфоцитов. Система комплемента состоит из плазменных протеинов и мембранных рецепторов. Плазменные протеины взаимодействуют между собой тремя известными каскадными путями – лектиновым (наиболее филогенетически древним), альтернативным и классическим.

Лектины – общий термин протеинов, формирующих отдельное суперсемейство паттерн-распознающих рецепторов, способных к распознаванию и агрегации молекул олиго- и полисахаридной природы. Среди всех лектинов уникальными функциями формирования комплексов с углеводными компонентами микробной стенки обладают фиколины (общий домен – фибрионоген) и коллектины (общий домен – коллаген) – маннозосвязывающий лектин (mannose-binding lectin, MBL), печеночный и почечный коллектины (Kilpatrick, 2002; Zelensky, Gready, 2005; Bjarnadottir et al., 2016; Troldborg et al., 2017). Образование сложного комплекса полисахариды микробной стенки+ коллектин/фиколин+специфические протеазы приводит в итоге к активации системы комплемента, воспалительной реакции и элиминации бактерии. Такой путь активации называется лектиновым.

MBL – классический лектин С-типа (C-type lectin), состоящий из нескольких субъединиц и склонный к олигомеризации до димеров, тримеров и тетрамеров. Способность к олигомеризации генетически детерминирована и критически повышает активность MBL в отношении связывания полисахаридов бактерий и активации комплемента (Kilpatrick, 2002). В настоящее время известно, что доминантные мутации в экзоне 1 гена MBL, распо-ложенного на хромосоме 10 (10q21.1), приводят к снижению способности MBL к олигомеризации и, следовательно, к снижению его плазменной концентрации и функциональной активности. К таким однотипным последствиям приводят мутации в кодонах 52 (rs5030737; A/D), 54 (rs1800450; A/B) и 57 (rs1800451; A/C). Аллели, содержащие мутации в кодонах 52, 54 и 57, обозначают как D, B и C соответственно, в отличие от дикого аллеля A. Всвязи с однотипными физиологическими последствиями мутации D, B и C принято объединять и обозначать O.

Кроме кодирующих мутаций в экзоне 1, на иммунологическую функцию MBL также влияют мутации в промоторе гена: диморфизмы в локусах rs11003125 (H/L) и rs7096206 (Y/X) модулируют транскрипционную активность, значительно влияя на концентрацию MBL в плазме крови (H > L и Y > X) (Kilpatrick, 2002). Установлено, что HY диплотип ассоциирован с наиболее высокой плазменной концентрацией MBL, LY диплотип – со средней, а LX – с низкой (Madsen et al., 1995). Кроме того, был выявлен диморфизм в некодирующем регионе экзона 1 (rs7095891; P/Q). 

В связи с выраженным неравновесным сцеплением все описанные мутации могут комбинироваться в ограниченное число гаплотипов из 64 возможных (HYPA, LXPA, LYQA, LYPA, HYPD, LYPB, LYPD и LYQС) (Madsen et al., 1995; Sullivan et al., 1996). Распределение частот гаплотипов гена MBL имеет крайне выраженные популяционные различия (Madsen et al., 1995; Boldt et al., 2006). Так, частота встречаемости гаплотипа HYPA, ассоциированного с высокой концентрацией MBL, варьирует от 6–8 % в африканских популяциях – Мозамбик, Кения (Madsen et al., 1995, 1998) – до 64–81 % в северных коренных популяциях – североамериканские индейцы и инуиты (Hegele et al., 1999; Best et al., 2004; Monsey et al., 2019). Европеоиды в этой градации занимают промежуточное положение с 27–30 % частотой гаплотипа HYPA (Skalnikova et al., 2004; Bernig et al., 2005; Steffensen et al., 2000).

Дополнительно, для оценки клинических последствий генетически детерминированных различий в экспрессии MBL было предложено выделять MBL-дефицитные (YO/YO или XA/YO), MBL-промежуточные (YA/YO или XA/XA) и MBL-высокоэкспрессирующие (YA/YA или XA/YA) диплотипы (Garred et al., 2009; Monsey et al., 2019). Принято считать, что 20–25 % всей человеческой популяции являются носителями MBL-дефицитных гаплотипов, а у 8–10 % MBL в плазме крови отсутствует или крайне низок (Madsen et al., 1995; Chalmers et al., 2013; Eisen, Osthoff, 2014).

Большинство MBL-дефицитных индивидов в целом здоровы. Явные клинические последствия MBL-дефицит имеет только в отдельных клинических ситуациях: у пациентов с нейтропенией, после трансплантации органов и тканей, у новорожденных, особенно у недоношенных (Luo et al., 2014; Czerewaty et al., 2019). В то же время значительное количество исследований показывает, что генетически детерминированный уровень MBL может модифицировать риск возникновения и клинические характеристики многих инфекционных заболеваний. Такое влияние имеет плюрипотентный характер.

Высокий уровень MBL является протективным фактором в отношении возникновения и тяжести инфекций, вызванных инкапсулированными бактериями (Streptococcus pneumoniae, Haemophilus influenzae и Neisseria meningitidis), прежде всего у детей раннего возраста (Eisen et al., 2008; Tereshchenko et al., 2016). В то же время была высказана гипотеза, что нормальные/высокие уровни MBL могут повышать риск инфицирования и избыточной воспалительной реакции при инфекциях, вызванных некоторыми внутриклеточными возбудителями (Mycobactеrium tuberculosis, Leishmania) (Verdu et al., 2006; Eisen, Osthoff, 2014). Следовательно, носители некоторых MBL-дефицитных гаплотипов могут иметь определенное клиническое преимущество при этих внутриклеточных инфекциях. Последние проведенные метаанализы показывают, что связь MBL генотипов с туберкулезом неоднозначна: некоторые генетические вариации повышают риск заболевания (rs1800450, rs5030737), а некоторые могут его снижать (rs1800451, rs7095891) (Areeshi et al., 2016; Cao et al., 2018; Tong et al., 2019). Анализ осложняет большая гетерогенность клинических форм туберкулеза в проведенных исследованиях. К тому же оценка риска в значительной мере может зависеть от этнического и возрастного состава исследованных популяций (Areeshi et al., 2016; Cao et al., 2018; Zhang et al., 2020). Насколько нам известно, к настоящему времени не опубликованы данные относительно распределения генотипов и гаплотипов гена MBL2 в русской популяции Восточной Сибири и у коренных жителей, проживающих на территориях Арктической зоны Российской Федерации.

## Материалы и методы

Для изучения однонуклеотидных полиморфизмов гена MBL2 в Красноярском краевом консультативно-диагностическом центре медицинской генетики было получено в общей сложности 880 образцов высохших пятен крови от новорожденных из Таймырского Долгано-Ненецкого района Красноярского края. Материалом исследования послужила ДНК, выделенная из периферической крови с использованием набора DIAtom DNAPrep100 (ООО «Изоген», Россия). Новорожденные были разделены на четыре группы для изучения этнической специфики полиморфизмов MBL2:

- 1 – 260 человек из деревень с преимущественно ненецким населением (ненцы составляют 85 % населения);
- 2 – 110 человек из деревень с преимущественно долган-нганасанским населением (долганы-нганасаны составляют 91 % населения);
- 3 – 210 человек из деревень со смешанным населением с различной комбинацией коренных и смешанных популяций;
- 4 – 300 новорожденных из города Красноярска, имеющих европейские корни (русские составляют 91 % населения).

Исследование было одобрено этическим комитетом Научно-исследовательского института медицинских проблем Севера (№ 9 от 8.09.2014). Получено письменное информированное согласие на проведение исследования от родителей.

Генотипирование двух полиморфизмов rs1800450 и rs1800451 произведено с помощью рестрикционного анализа продуктов амплификации (ПДРФ-анализ) специфических участков генома. Фрагмент из 349 bp был амплифицирован с использованием пары праймеров: forward 5′-TAGGACAGAGGGCATGCTC-3′ и reverse 5′-CA GGCAGTTTCCTCTGGAAGG-3′ (температура отжига 60 °C). Эндонуклеазы рестрикции AccB1 I (rs1800450) и Mbo II (rs1800451) применяли для гидролиза амплификатов и далее фрагменты разделяли в 2 % агарозном геле с этидиумом бромидом для визуализации результатов. В случае rs1800450 полиморфизма использовали рестриктазу AccB1 I: фрагмент 349 bp соответствовал B аллелю, а два фрагмента 260 и 89 bp – A аллелю. В случае rs1800451 использовали Mbo II эндонуклеазу: фрагмент 349 bp соответствовал A аллелю, а два фрагмента 270 и 79 bp – C аллелю.

Генотипирование однонуклеотидных полиморфизмов MBL2 rs11003125, rs7096206, rs7095891 и rs5030737 осуществлено при помощи метода ПЦР в режиме реального времени с использованием специфических олигонуклеотидных праймеров и флуоресцентно-меченных зондов (TagMan) (ООО «ДНК-синтез», Россия) по протоколу производителя (табл. 1)

**Table 1.**
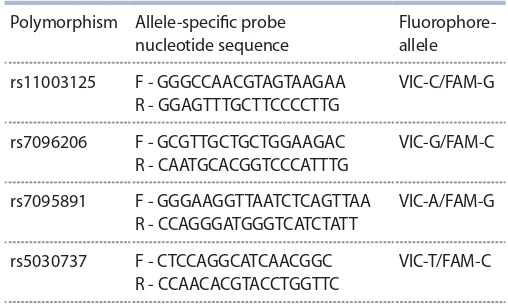
Nucleotide sequences of allele-specific probes for genotyping

Соответствие частот генотипов равновесию Харди – Вайнберга проверено с использованием χ2. Сравнения частот генотипов проводили с использованием точного двустороннего теста Фишера. Гаплотипы оценивали и сравнивали между популяциями с использованием пакета haplo.stats для R среды. Для множественного тестирования применена коррекция Бонферрони. Статистически значимые различия были приняты при р < 0.05 после коррекции для множественного тестирования.

## Результаты и обсуждение

Частоты генотипов всех включенных в исследование полиморфных участков гена MBL2, за исключением rs1800451, представлены в табл. 2. 

**Table 2.**
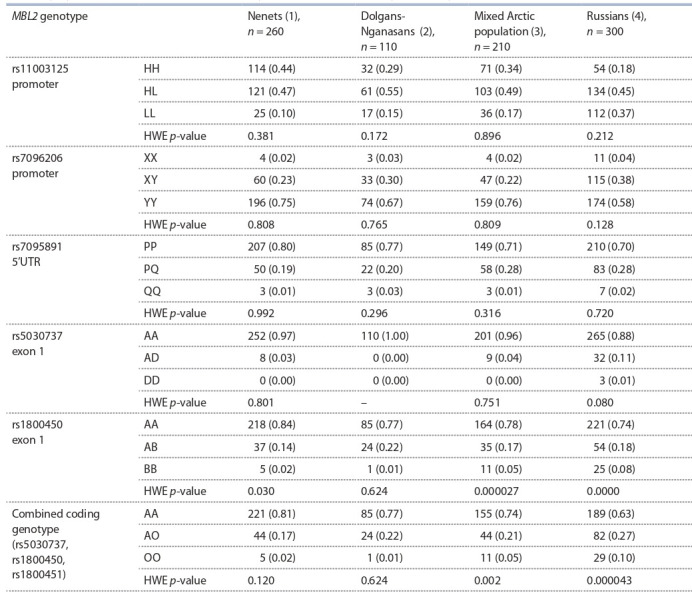
MBL2 genotype frequencies among newborns from different ethnic populations of the Taymyr Dolgan-Nenets raion of the Krasnoyarsk oblast and of the city of Krasnoyarsk, n (%)

Вариантный аллель С в участке rs1800451 обнаружен только в одном случае из 880 протестированных новорожденных – в гомозиготном состоянии (СС) у европеоида, проживающего в Красноярске. Среди гомозиготных вариаций изученных полиморфизмов гена MBL2 наиболее заметные популяционные различия выявлены в промоторном регионе для участка rs11003125, где частота генотипа LL, ассоциированного с низкой продукцией MBL, в русской популяции превышала частоты в коренных популяциях Арктики в 2–3 раза: русские – 37 %, ненцы – 10 %, долганы-нганасаны – 15 % ( p1–2, 3 < 0.001).

Комбинированный аллель O был рассчитан на основании анализа мутаций в кодонах 52 (rs5030737, A/D), 54 (rs1800450, A/B) и 57 (rs1800451, A/C). Как указано выше, аллели, содержащие мутации в кодонах 52, 54 и 57, обозначены как D, B и C соответственно, в отличие от дикого аллеля A. Мутации D, B и C были закодированы и обозначены O. Частота комбинированного редкого аллеля О, он сформирован из кодирующих участков rs5030737, rs1800450 и rs1800451, в гомозиготном состоянии также была значительно выше в популяции русских новорожденных: русские – 10 %, ненцы – 2 %, долганы-нганасаны – 1 % ( p1–2, 3 < 0.001).

Наши данные о частоте гаплотипов гена MBL2 показывают, что частота высокопродуцирующего гаплотипа HYPA составляет 35.4 % у русских новорожденных Восточной Сибири (табл. 3). Это соответствует частотам европейских популяций: Голландии – 27 % (Bernig et al., 2005), Дании – 30 % (Steffensen et al., 2000), Чехии – 33 % (Skalnikova et al., 2004), а также европеоидов Бразилии – 28–34 % (Boldt et al., 2006; Ferraroni et al., 2012). В то же время у новорожденных арктических популяций частота гаплотипа HYPA была статистически значимо выше, чем у русских, и составила 64 % для ненцев и 56 % для долган-нганасан, что приближается к значениям частот распространения, выявленных для эскимосов, – 81 % (Madsen et al., 1995; Hegele et al., 1999) и североамериканских индейцев – 64 % (Best et al., 2004). Одновременно у новорожденных российских арктических популяций закономерно зарегистрированы низкие частоты MBL-дефицитного гаплотипа LXPA (см. табл. 3). Наибольшие различия в частотах указанных гаплотипов были характерны для ненецкой популяции.

**Table 3.**
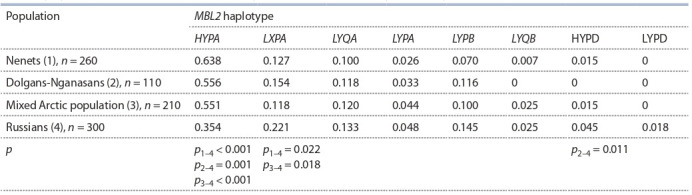
MBL2 haplotype frequencies among newborns from different ethnic populations of the Taymyr Dolgan-Nenets raion of the Krasnoyarsk oblast and of the city of Krasnoyarsk Notе. Only p < 0.05 values are shown in the table; when calculating the p-value, corrections were made for multiple comparisons.

В табл. 4 суммированы данные о частотах MBL-дефицитных гаплотипов в изученных популяциях. Выделены MBL-дефицитные (YO/YO или XA/YO), MBL-промежуточные (YA/YO или XA/XA) и MBL-высокоэкспрессирующие (YA/YA или XA/YA) гаплотипы. 

**Table 4.**
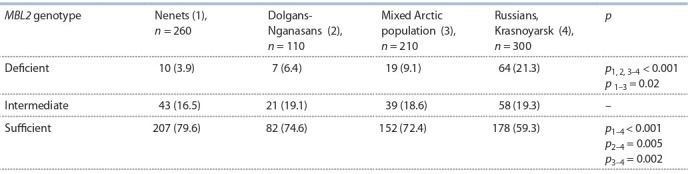
The prevalence of MBL-deficient haplotypes among newborns from different ethnic populations of the Taymyr Dolgan-Nenets raion of the Krasnoyarsk oblast and of the city of Krasnoyarsk, n (%)

Популяции ненцев и долган-нганасан демонстрируют существенно более низкие частоты MBL-дефицитных гаплотипов в сравнении с европеоидами Восточной Сибири (3.9, 6.4 и 21.3 % соответственно, p < 0.001). Смешанная арктическая популяция демонстрирует промежуточное значение частоты – 9.1 %. На популяционном уровне клинические последствия врожденно высокой способности продукции функционально активных форм MBL у представителей арктических популяций заключаются в низком риске тяжелых бактериальных инфекций в раннем возрасте и, вероятно, более высоком риске туберкулеза в старшем возрасте, что предполагается многими исследователями (Eisen, Osthoff, 2014; Tong et al., 2019). Кроме того, низкая частота атеросклероза и кардиоваскулярных заболеваний среди коренных жителей Арктики, наряду с такими факторами, как высокое употребление омега-3 жирных кислот и особенности стиля жизни, может быть обусловлена и генетическими особенностями продукции и активности MBL. Вероятность такой связи показана в целом ряде публикаций (Hegele et al., 1999; Best et al., 2004; Fumagalli et al., 2017; Monsey et al., 2019).

В настоящем исследовании нами впервые получены данные о частотах генотипов и гаплотипов гена MBL2 среди коренных народностей, проживающих на территориях Арктической зоны Российской Федерации. Ранее нами была показана большая частота распространенности генотипов, ассоциированных с высокой активностью L-фиколина, в арктических популяциях ненцев и долганнганасан, в сравнении с европеоидами Восточной Сибири (Smolnikova et al., 2017). Таким образом, популяции коренных народов Арктики генетически характеризуются большей активностью как минимум двух различающихся компонентов лектинового пути активации комплемента – MBL и L-фиколина. Определенное преимущество нашего подхода к популяционной оценке распространенности MBL- и L-фиколин генотипов состоит в исследовании популяций новорожденных, когда еще не произошло вероятное выбывание неблагоприятных генетических вариаций, возможное в более старшем возрасте. 

В настоящее время существуют две конкурирующие гипотезы, пытающиеся объяснить высокий уровень популяционного разнообразия генотипов MBL2 с высоким накоплением MBL-дефицитных вариантов (Eisen, Osthoff, 2014).

Первая из них предполагает протективную роль низкопродуцирующих генотипов в отношении некоторых внутриклеточных возбудителей: туберкулеза и лепры (микобактерии), висцерального лейшманиоза (род внутриклеточных паразитов Leishmania), атипичной пневмонии (внутриклеточные бактерии Mycoplasma pneumoniae, Chlamydophila pneumoniae, Legionella pneumophila, Coxiella burnetii). Впервые предположение о роли позитивной селекции в накоплении низкопродуцирующих генотипов высказано P. Garred и его сотрудниками в 1994 г. Они установили, что у пациентов с лепрой (возбудитель Mycobacterium leprae) уровень MBL в сыворотке крови был выше, чем у здоровых доноров той же популяции (Garred et al., 1994). В 1999 г. E.G. Hoal-Van Helden с коллегами показали протективную роль MBL-низкопродуцирующего аллеля В полиморфного участка rs1800450 гена MBL в отношении туберкулезного менингита (Hoal-Van Helden et al., 1999). Последние проведенные метаанализы также подтверждают роль полиморфизма MBL2 при формировании туберкулезной инфекции (Areeshi et al., 2016; Cao et al., 2018; Tong et al., 2019). В 2001 г. I.K. Santos с сотрудниками показали, что MBL-низкопродуцирующий вариантный генотип OO встречался реже у пациентов с висцеральным лейшманиозом (Santos et al., 2001). В дальнейшем эти данные были подтверждены: риск висцерального лейшманиоза был значительно повышен у лиц с генетическими вариантами, ассоциированными с высокой продукцией MBL (Alonso et al., 2007). Наконец, недавнее проспективное исследование датской когорты пациентов с внебольничной пневмонией (n = 505) показало большую предрасположенность лиц с генетической детерминированной высокой продукцией основных факторов лектинового пути активации комплемента MBL и L-фиколина к внутриклеточным респираторным инфекциям: Mycoplasma pneumoniae, Chlamydophila pneumoniae, Legionella pneumophila, Coxiella burnetii (Van Kempen et al., 2017). Большинство исследователей считают, что высокий уровень лектин-опосредованного фагоцитоза может предрасполагать к более успешному проникновению внутриклеточных возбудителей в цитоплазму клеток хозяина, экранированию патогенов от факторов адаптивного иммунитета и, следовательно, большему риску формирования активного инфекционного процесса

Кроме того, в ряде работ показано, что MBL-дефицит может быть протективным фактором в отношении атеросклероза и ассоциированных кардиоваскулярных заболеваний (Hegele et al., 1999; Best et al., 2004; Fumagalli et al., 2017; Monsey et al., 2019). Секвенирование генома 102 жителей США, представителей четырех основных этнических групп, показало наличие признаков селективного отбора в сторону большего накопления гетерозигот гена MBL2 (Bernig et al., 2004). 

Имеющиеся к настоящему времени фактические данные позволяют говорить о «двойной патофизиологической» роли лектинового пути активации комплемента: протективной – в отношении внеклеточных возбудителей, особенно у детей раннего возраста, и провокативной – в отношении некоторых внутриклеточных возбудителей и атеросклероза. Популяционно-генетические последствия такой «двойной» роли могут лежать в основе этнического разнообразия соответствующих генотипов, что представляет собой суть первой упомянутой нами гипотезы, основанной на предположении селекционной выгоды MBL-дефицита для некоторых популяций (Seyfarth et al., 2005; Eisen, Osthoff, 2014). В русле указанной гипотезы было высказано предположение, объясняющее низкую частоту MBL-дефицита среди арктических народностей и генетически близких к ним коренных североамериканских индейцев. Принято считать, что эти популяции исторически позже встретились с туберкулезом и лепрой, не сталкивались с возбудителями лейшманиоза и реже имели классические факторы риска атеросклероза: диабет, гиперлипидемию, а также, возможно, хроническое инфицирование Chlamydophila pneumoniae (Hegele et al., 1999; Best et al., 2004; Monsey et al., 2019). Следовательно, именно в этих популяциях не происходила характерная, согласно этой гипотезе, позитивная селекция MBL-дефицитных генотипов

Вторая гипотеза отрицает наличие селекционного давления в отношении MBL2 генотипов, объясняя генетическое разнообразие исключительно миграционными процессами и генетическим дрейфом. Так, исследование 1116 индивидов из различных географических регионов не выявило статистических признаков селективного отбора (Verdu et al., 2006). Такие же результаты были получены при статистической обработке данных различных популяций Бразилии и сравнительном изучении жителей Габона и Европы (Boldt et al., 2006, 2010). Исследование MBL2 полиморфизма у детей Мозамбика показало отсутствие статистических признаков позитивной или балансирующей селекции (Valles et al., 2009). Впрочем, некоторые авторы делают при обсуждении собственных результатов оговорку: «Возможно, стохастические эволюционные факторы стерли большую часть древнего отпечатка, оставленного естественным селекционным отбором; для подтверждения данных требуются статистически более мощные исследования с включением большего числа популяций» (Boldt et al., 2006).

## Заключение

Таким образом, по результатам настоящего исследования нами показана большая частота встречаемости MBL-высокопродуцирующих генетических вариаций в популяциях коренных арктических народностей, проживающих в Таймырском Долгано-Ненецком районе Красноярского края. Рассматривая представленные данные в совокупности с ранее опубликованными результатами полиморфизма гена L-фиколина в тех же популяциях (Smolnikova et al., 2017), можно говорить не только о накоплении отдельных генотипов MBL2 и FCN2 в арктических популяциях, но и о большем тонусе лектинового пути активации комплемента в целом. Указанные факты позволяют нам осторожно высказаться о гипотезе селективного популяционного давления в отношении лектинового пути активации комплемента как общего патофизиологического механизма, опосредованного генами MBL2 и FCN2, и, вероятно, ассоциированного с предрасположенностью к некоторым инфекциям. Мы считаем, что изолированные арктические популяции исторически позже столкнулись с некоторыми внутриклеточными инфекциями (микобактериями, возможно Chlamydophila pneumoniae) и вследствие этого сохранили сформированную на ранних этапах эволюции человека высокую активность лектинового пути активации комплемента. Безусловно, эта гипотеза требует дополнительной верификации в специально организованных исследованиях большей статистической мощности с использованием всего арсенала методов популяционной генетики.

## Conflict of interest

The authors declare no conflict of interest.
